# Waltzing around cofactors

**DOI:** 10.7554/eLife.13977

**Published:** 2016-02-03

**Authors:** Percival Yang-Ting Chen, Elizabeth C Wittenborn, Catherine L Drennan

**Affiliations:** 1 Department of Chemistry, Massachusetts Institute of Technology, Cambridge, United States; 1 Department of Chemistry, Massachusetts Institute of Technology, Cambridge, United States; 2 Departments of Chemistry and Biology and the Howard Hughes Medical Institute, Massachusetts Institute of Technology, Cambridge, United Statescdrennan@mit.edu

**Keywords:** nitrogen fixation, metalloproteins, Azotobacter vinelandii, iron sulfur proteins, Other

## Abstract

The metallocofactor involved in fixing nitrogen is not a rigid scaffold, as was previously thought.

**Related research article** Spatzal T, Perez KA, Howard J, Rees DC. 2015. Catalysis-dependent selenium incorporation and migration in the nitrogenase active site iron-molybdenum cofactor. *eLife* **4**:e11620. doi: 10.7554/eLife.11620

**Image** A FeMo-cofactor in which one of the "belt" sulfur atoms has been replaced by a selenium atom (green)

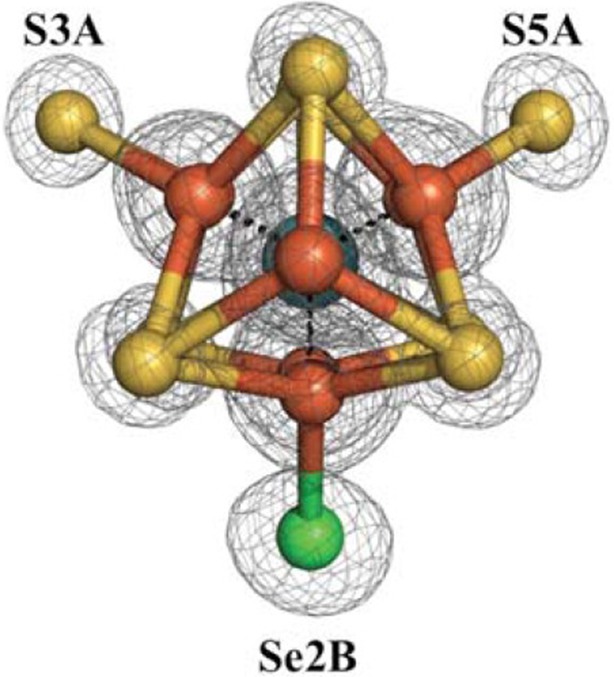

Nitrogen is an element that is found in nearly all important biological molecules, including amino acids and nucleic acids. Nitrogen is also abundant in our atmosphere in the form of N_2_ molecules, but this form of the element cannot be used biologically, so N_2_ must be transformed or “fixed” into biologically reactive compounds such as ammonia (NH_3_) and nitrate (NO_3_^-^).

Nature employs enzymes called nitrogenases to fix nitrogen. These enzymes rely on “cofactors” to perform their chemistry: the most widely studied of these cofactors contains iron and molybdenum, and hence is called the FeMo-cofactor (Figure 1). Despite the fact that nitrogenase activity was discovered in 1934, a comprehensive understanding of how it works has remained elusive due to the intrinsic complexity of both the FeMo-cofactor and the nitrogen fixation reaction (Hoffman et al., 2014). Now, in eLife, Thomas Spatzal, Kathryn Perez, James Howard and Douglas Rees report that some of the atoms that make up the FeMo-cofactor migrate in unexpected ways during catalysis (Spatzal et al., 2015).

**Figure 1.**
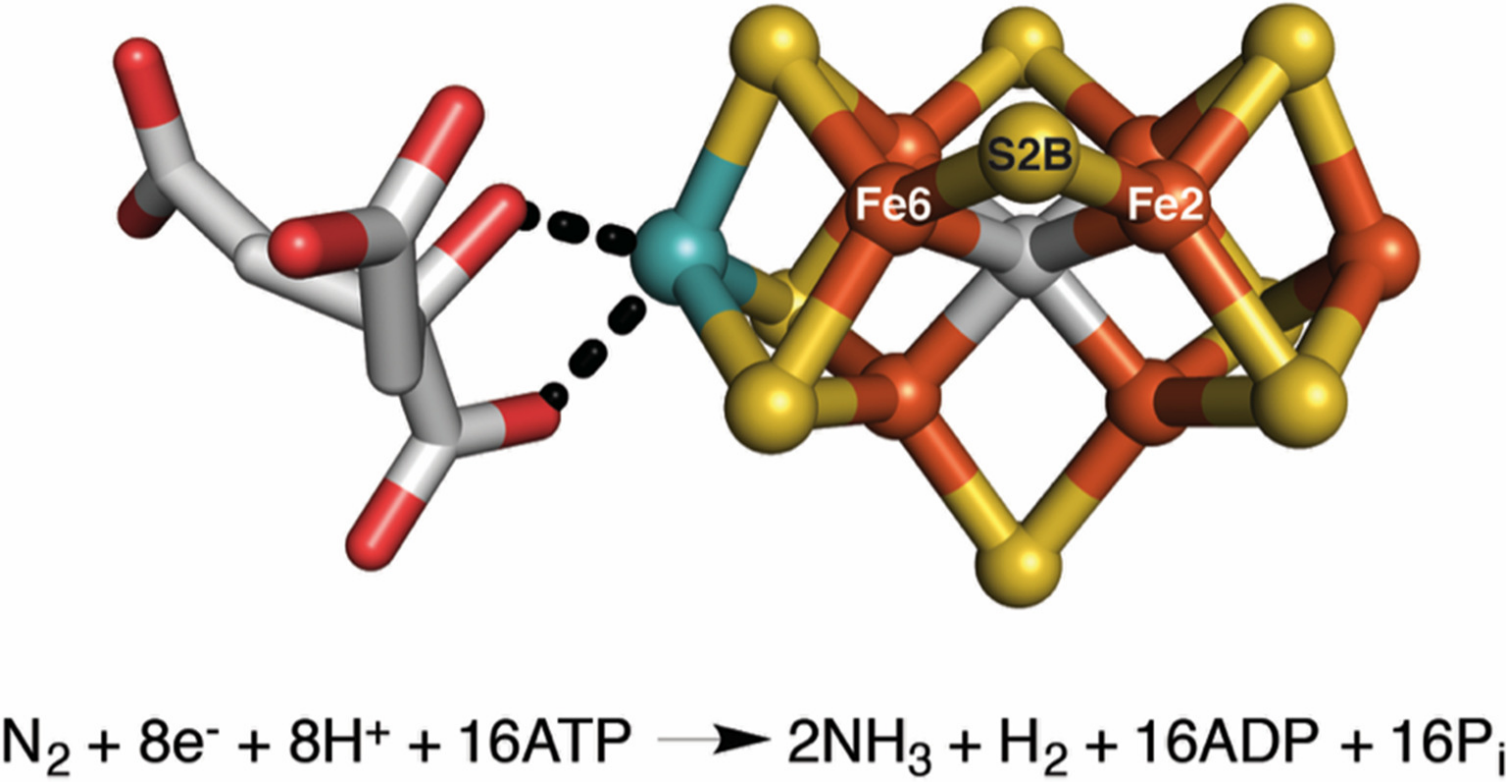
The FeMo-cofactor contains a [7Fe-9S-C-Mo] center (right) coordinated to (*R*)-homocitrate (left) (Kim and Rees, 1992; Einsle et al., 2002; Spatzal et al., 2011). Spatzal, Perez et al. were able to insert a selenium atom into the S2B position of the FeMo-cofactor, and track how it moves during catalysis. Iron (Fe) atoms are shown in orange, sulfur (S) atoms in yellow, carbon in gray, molybdenum in cyan, and oxygen in red. The overall reaction is also shown. Reduction of each nitrogen molecule (N_2_), requires eight electrons and at least eight protons (H^+^), yielding two ammonia molecules (NH_3_) and one hydrogen molecule (H_2_). This nitrogen fixation reaction is coupled to the hydrolysis of 16 adenosine triphosphate (ATP) molecules (Burgess and Lowe, 1996; Howard and Rees, 2006; Hoffman et al., 2014).

Molybdenum nitrogenase is an enzyme that contains a molybdenum-iron protein (which is catalytic) and an iron protein (which is involved in electron transfer). The molybdenum-iron protein is home to the FeMo-cofactor and a cluster of iron and sulfur atoms that transfers electrons between the iron protein and the FeMo-cofactor. During the reaction cycle, the FeMo-cofactor orchestrates the reduction of two distinct substrates, N_2_ and H^+^, and allocates hydrogen atoms into products (NH_3_ or H_2_). Elucidating a detailed mechanism for this process is important for understanding how molybdenum nitrogenase is able to achieve this challenging chemical transformation.

To clarify how substrates bind to the FeMo-cofactor, Rees and co-workers previously solved a high-resolution X-ray crystal structure of the molybdenum-iron protein bound to carbon monoxide, a molecule that inhibits the cofactor (Spatzal et al., 2014). Unexpectedly, this structure revealed that carbon monoxide replaces the sulfur atom at the S2B position in the FeMo-cofactor; this atom is one of three "belt sulfurs" that form bridges between pairs of iron atoms in the cofactor (Figure 1). Significantly, this process is reversible; reactivated molybdenum-iron protein was shown to once again contain sulfur in the S2B position. These structures serve to shed light on substrate binding in molybdenum nitrogenase and suggest a previously unanticipated kinetic instability, or lability, of the FeMo-cofactor.

Inspired by this apparent lability of the FeMo-cofactor, Spatzal, Perez et al. – who are based at the California Institute of Technology and the University of Minnesota – have now developed a means of labeling the S2B site of the FeMo-cofactor with selenium, the element immediately below sulfur in the periodic table. Selenocyanate is a compound that contains selenium, and has recently been identified as a substrate and inhibitor of nitrogenase. Spatzal, Perez et al. therefore used X-ray crystallography to identify changes in the molybdenum-iron protein after nitrogenase had reacted with selenocyanate. In particular, they used the fact that selenium scatters X-rays differently from sulfur to confirm that selenium replaces sulfur at the S2B position, further strengthening the notion that S2B is a labile element in the FeMo-cofactor.

Spatzal, Perez et al. then showed that incorporating selenium into the FeMo-cofactor does not alter the catalytic activity of the enzyme. Taking a series of crystallographic snapshots of the selenium-containing cofactor at different stages of catalysis revealed that under turnover conditions, selenium migrates to the other two belt-sulfur positions. Selenium is then replaced by sulfur after reacting with multiple substrates. However, this migration of selenium is only observed when N_2_ or acetylene (C_2_H_2_) are used as substrates, and not when H^+^ is the exclusive substrate.

Before this work, the dogma in the field was that the atoms in the FeMo-cofactor and other metallocofactors provide a rigid scaffold on which chemistry can be performed, with movements restricted to protein sidechains, substrates and products. Now we must consider that in nitrogenase – and potentially other enzymes – the atoms of metallocofactors may actively change positions and that this dynamic nature may be substrate-specific.

The work of Spatzal, Perez et al. raises many additional questions. For example, how does belt-sulfur migration correlate with the reaction mechanism? And how are the structural dynamics of the FeMo-cofactor coupled to the reduction of different substrates? It also provides a powerful tool for interrogating the FeMo-cofactor through site-specific selenium incorporation. Already this method has allowed us to see selenium waltzing around the FeMo-cofactor center, and it is hard to imagine what surprises this enzyme has in store for us next.
